# Design of a Nasal Spray Based on *Cardiospermum halicacabum* Extract Loaded in Phospholipid Vesicles Enriched with Gelatin or Chondroitin Sulfate

**DOI:** 10.3390/molecules26216670

**Published:** 2021-11-04

**Authors:** Eleonora Casula, Maria Manconi, José Antonio Vázquez, Tania Belen Lopez-Mendez, José Luis Pedraz, Esteban Calvo, Antonio Lozano, Marco Zaru, Andreia Ascenso, Maria Letizia Manca

**Affiliations:** 1 Department of Scienze della Vita e dell’Ambiente, Sezione di Scienze del Farmaco, University of Cagliari, Via Ospedale n. 72, 09124 Cagliari, Italy; e.casula@studenti.unica.it (E.C.); mlmanca@unica.it (M.L.M.); 2 Group of Recycling and Valorization of Waste Materials (REVAL), Marine Research Institute (IIM-CSIC), C/Eduardo Cabello, 6, 36208 Vigo, Spain; jvazquez@iim.csic.es; 3 NanoBioCel Group, Laboratory of Pharmaceutics, School of Pharmacy, University of the Basque Country (UPV/EHU), Paseo de la Universidad 7, 01006 Vitoria-Gasteiz, Spain; tania.lopez@ehu.eus (T.B.L.-M.); joseluis.pedraz@ehu.eus (J.L.P.); 4 Biomedical Research Networking Center in Bioengineering, Biomaterials and Nanomedicine (CIBER-BBN), 01006 Vitoria-Gasteiz, Spain; 5 Bioaraba, NanoBioCel Research Group, Calle José Achotegui s/n., 01009 Vitoria-Gasteiz, Spain; 6 Laboratory for Research in Fluid Dynamics and Combustion Technology (LIFTEC), Consejo Superior de Investigaciones Cientificas (CSIC)–Universidad de Zaragoza, María de Luna, 10, 50018 Zaragoza, Spain; Esteban.Calvo@unizar.es (E.C.); a.lozano@csic.es (A.L.); 7 Icnoderm S.r.l., Sardegna Ricerche Ed. 5, Pula, 09010 Cagliari, Italy; m.zaru@icnoderm.com; 8 Faculty of Pharmacy, University of Lisbona, Av. Gama Pinto, 1649-003 Lisbona, Portugal; andreiaascenso@ff.ulisboa.pt

**Keywords:** *Cardiospermum halicacabum*, epithelial cells, droplet size distribution, plume angle, antioxidant activity, keratinocytes

## Abstract

The extract of *Cardiospermum halicacabum* L. (*C. halicacabum*) obtained from flower, leaf and vine was loaded into modified phospholipid vesicles aiming at obtaining sprayable, biocompatible and effective nasal spray formulations for the treatment of nasopharyngeal diseases. Penetration enhancer-containing vesicles (PEVs) and hyalurosomes were formulated, and stabilized by adding a commercial gelatin from fish (20 mg/mL) or chondroitin sulfate from catshark cartilages (*Scyliorhinus canicula,* 20 mg/mL). Cryo-TEM images confirmed the formation of spherical vesicles, while photon correlation spectroscopy analysis disclosed the formation of small and negatively-charged vesicles. PEVs were the smaller vesicles (~100 nm) along with gelatin-hyalurosomes (~120 nm), while chondroitin-PEVs and chondroitin-hyalurosomes were larger (~160 nm). Dispersions prepared with chondroitin sulfate were more homogeneous, as the polydispersity index was ~0.15. The in vitro analysis of the droplet size distribution, average velocity module and spray cone angle suggested a good spray-ability and deposition of formulations in the nasal cavity, as the mean diameter of the droplets was in the range recommended by the Food and Drug Administration for nasal targets. The spray plume analysis confirmed the ability of PEVs, gelatin-PEVs, hyalurosomes and gelatin-hyalurosomes to be atomized in fine droplets homogenously distributed in a full cone plume, with an angle ranging from 25 to 30°. Moreover, vesicles were highly biocompatible and capable of protecting the epithelial cells against oxidative damage, thus preventing the inflammatory state.

## 1. Introduction

*Cardiospermum halicacabum* L. (*C. halicacabum*) belongs to the Sapindaceae family, which includes about 1200 species commonly found in India, South America and Africa. The beneficial properties of *C. halicacabum* have been known for centuries as a natural cortisone for the treatment of rheumatism, as well as a remedy for digestive and respiratory disorders, joint and back pain and muscle sprains, and also as a product capable of contrasting the poisoning effect of snake bites [1,2,3,4,5,6,7,8,9,10]. The extract of *C. halicacabum* is still used in traditional eastern medicine as an anti-inflammatory remedy against rhinopharyngitis, but the effect has never been evaluated in scientific studies. The main bioactives contained in the plant are flavonoids, triterpenoids, glycosides, fatty acids and volatile esters [11]. They synergically exert anti-inflammatory effects, reducing the activity of phospholipases A2, with a consequent reduction of the availability of arachidonic acid, a precursor of prostaglandin biosynthesis [12]. At the same time, they may suppress an inflammatory mediator, such as nitrite oxide and cytokines such as TNF-α and free radical species, which are usually generated during the inflammatory response and in excess can potentiate and prolong the tissue damages [13]. Moreover, *C. halicacabum* counteracts free radicals, due to its content of powerful antioxidants including flavonoids, saponins, tannins, and glycosides [4,14].

Considering the promising anti-inflammatory and antioxidant power of this plant, its formulation in a nasal spray should represent an optimal alternative to effectively reach the nasal cavity for the treatment of nasal congestion and rhinitis, especially if associated with a suitable nasal preparation with a moisturizing effect, capable of counteracting the dryness and crusting usually associated with these disorders [15]. Phospholipid vesicles seem to be the ideal systems for this propose, because they are used on the inflamed nasal mucosa to alleviate the symptoms of seasonal allergic rhinitis [16,17]. Currently, a liposome-based spray is commercially available in Germany and a formulation containing hyalurosomes (phospholipid vesicles immobilized with hyaluronic acid) is present on the Italian market [18].

The combination of the extract of *C. halicacabum* with the phospholipid vesicles in a nasal spray represents a promising strategy for the local treatment of nasal congestion and rhinitis [15,19,20,21,22,23,24]. Moreover, *C. halicacabum* extract was not previous loaded in phospholipid vesicles and the positive effects provided by this association has not explored previously, along with their suitability as nasal spray. Indeed, to provide a local effect, nasal sprays must have specific features, such as adequate spray plume and droplet size distribution, which depend on the properties of the pump, the formulation, the orifice of the actuator, and the force applied [25]. These parameters can positively affect the deposition zone in the nose, improving the beneficial effect of the bioactives at a local level, by enhancing their accumulation and efficacy in the anterior nose [15,25,26]. Polymers are widely added in nasal sprays to improve the bioadhesiveness and mucosa dehydrations, as a function of their specific properties. Gelatin is widely used over other polymers in nanotechnology thanks to its high biocompatibility, biodegradability and non-irritability. Combined with liposomes, gelatin improves the stability of the final formulations because of its capacity to adsorb and retain water and reduce the water vapor permeability, slowing the release time of the drugs [27,28]. Chondroitin sulfate is a constituent of proteoglycans—safe, biocompatible and widely used to produce biomaterial and biomedical devices [29,30,31]. Research has highlighted various pharmacological properties of chondroitin sulfates from *Scyliorhinus canicula*, such as antithrombotic [29], anticoagulant [30], antioxidant, anti-inflammatory and neuroprotective activities [31].

Considering the lack of knowledge in the present study, the glycolic extract of *C. halicacabum* was loaded in penetration enhancer-containing vesicles (PEVs) and hyalurosomes, which were further modified by adding a commercial gelatin from cold-water fish or a biopolymer chondroitin sulfate, extracted from catshark cartilages of *Scyliorhinus canicular* [32,33,34,35]. The spray-ability, biocompatibility and effectiveness of vesicles as a nasal spray was evaluated. The mean size and zeta potential of vesicles were measured by a dynamic laser light scattering, and the aptitude to be sprayed in the nose (i.e., droplet size distribution, average droplet velocity and spray plume angle) was investigated by laser diffraction technique, laser plane visualization and particle image velocimetry. The cytotoxicity of the formulations and their ability to protect epithelial cells (keratinocytes) against oxidative stress were determined in vitro as well.

## 2. Materials and Methods

### 2.1. Materials 

Lipoid S75 (~70% of soy phosphatidylcholine, 9% phosphatidylethanolamine and 3% lysophosphatidylcholine) was purchased from Lipoid GmbH (Ludwigshafen, Germany). The 2:1 glycolic extract from flower, leaf and vine of *C. halicacabum* was kindly supplied by Sakura srl (Lonato del Garda-BS, Italy). Sodium hyaluronate with low molecular weight (200–400 kDa) and a polydispersity of 1.4 Mw/Mn was purchased from DSM Nutritional Products AG Branch Pentapharm (Rhein Elden, Switzerland). PEG400 was purchased from Galeno S.r.l. (Comeana, PO, Italy). Chondroitin sulfate was extracted from catshark cartilages (*Scyliorhinus canicula*) and kindly supplied by REVAL Institute (Vigo, Spain) [27,28,29]. The 2,2-diphenyl-1-picrylhydrazyl (DPPH), fish gelatin and all the other reagents of analytical grade were purchased from Sigma-Aldrich (Milan, Italy). Reagents and plastics for cell cultures were purchased from Life Technologies Europe (Monza, Italy).

### 2.2. Vesicle Preparation

For vesicle preparation, 90 mg of S75 and 3 mg of *C. halicacabum* extract was hydrated with 1 mL of a mixture of distilled water (900 µL/mL) and PEG400 (100 µL/mL) to obtain PEVs, or with 1 mL of dispersion of sodium hyaluronate (0.1%) in water (900 µL/mL) and PEG400 (100 µL/mL) to obtain hyalurosomes. In addition, PEVs and hyalurosomes were modified by adding gelatin (3 mg/mL, final concentration) or chondroitin sulfate (3 mg/mL, final concentration).

The dispersions were sonicated (25 cycles, 5 on/2 off) by using a Soniprep 150 sonicator (MSE Crowley, London, UK) to obtain a homogeneous dispersion with small vesicles. Empty vesicles without *C. halicacabum* extract were prepared as well and used as controls. Vesicles were stored at 4 °C.

### 2.3. Vesicle Characterization

The morphology of the vesicles has been evaluated by cryogenic transmission electron microscopy (cryo-TEM), by using a TECNAI G2 20 TWIN (FEI) microscope (FEI Company World Headquarters and North American Sales, 5350 NE Dawson Creek Drive Hillsboro, OR, USA), operating at an accelerating voltage of 200 KeV in a bright-field image mode and low-dose image mode. We applied 3 μL of sample dispersion to a glow-discharged 300 mesh Quantifoil TEM grid, which underwent plunge freezing into liquid ethane on a FEI Vitrobot Mark IV (Eindhoven, The Netherlands). The frozen grid was then transferred to a 626 DH Single Tilt Cryo-Holder (Gatan, France), maintained below −170 °C, and then transferred to TEM at the same temperature.

The average diameter, polydispersity index and zeta potential of each sample were determined by means of light scattering technique by using a Zetasizer Ultra (Malvern Panalytical Ltd., Malvern, UK). These parameters were also measured over a storage period of 7 months at 4 °C, to evaluate the long-term stability of vesicles.

Each sample (2 mL) was purified from the non-incorporated extract by dialysis (Spectra/Por^®^ membranes, 12–14 kD, 3 nm pore size; Spectrum Laboratories Inc., Rancho Dominguez, CA, USA) against water (2:1) at room temperature for 2 h and refreshing the water after 1 h. The antioxidant activity (AA) of the extract-loaded vesicles was measured by means of a 2-diphenyl-1-picrylhydrazyl (DPPH) colorimetric test, before and after the dialysis process. Twenty microlitres of each formulation was dissolved in 1980 µL of DPPH methanolic solution previously prepared (1:50). The methanolic solution of DPPH at the same dilution was used as a control (100% absorbance). Samples were incubated for 30 min in the dark at room temperature. Subsequently, the absorbance of each solution was measured at λ = 517 nm with a UV spectrophotometer (Lambda 25, Perkin Elmer, Milan, Italy). All the experiments were performed in triplicate. The antioxidant activity was calculated according to the following Formula (1) [36]:

(1) $$\mathrm{AA}\left(\%\right)=\frac{\left[\left({\mathrm{ABS}}_{\mathrm{DPPH}}-{\mathrm{ABS}}_{\mathrm{DPPH}}\right)\right]}{{\mathrm{ABS}}_{\mathrm{DPPH}}}\times 100$$

The entrapment efficiency (EE) of the vesicles was expressed as the percentage of the antioxidant activity before and after dialysis.

### 2.4. Spray Characterization

The velocity and angle of droplets generated by the nasal devices integrated with data provided by the measurement of droplet size distributions are essential parameters to evaluate the suitability of nasal spray formulations to evaluate their effective deposition in the nasal cavity and their correct administration to patients [37,38].

#### 2.4.1. Droplet Size Distribution

The size of the particles generated by spraying the formulations was measured by means of laser diffraction technique by using a Malvern Spraytec^®^ (Malvern Panalytical Ltd., Malvern, UK). For in vitro tests, the Food and Drug Administration (FDA) recommends determining the droplet size distribution at two distances comprised between 2 and 7 cm from the nozzle tip, with a difference of 3 cm apart [39,40,41]. Thus, herein the spray pattern data were obtained at two actuation distances from the laser beam, 4 and 7 cm. Twenty millilitres of each formulation were loaded into commercial spray pump devices kindly supplied by FAES Laboratories. The used devices were suitable for pharmacological applications composed of a 0.1 mL metered pump and a 30 mL spray bottle. Before each experiment, formulations were hand shaken for 10 s and then 3 actuations were shot blank to test the device. After applying a pressure on the supportive surface for fingers in the upper part of the device, the formulation inside was drawn up through a siphon tube from the bottom of the bottle and expelled through the nozzle [42].

Measurements were performed in triplicate at 25 °C, at 4 cm and 7 cm of distance and rotating the pump device 45° in relation to the laser beam. Data were reported as the D10, D50, and D90 volume diameter percentiles, expressed as 10%, 50% and 90% of the cumulative volume undersize. To characterize the width of droplet size distribution, the Span number was also reported as (D90−D10)/D50) as previously [43].

#### 2.4.2. Spray Structure, Velocity Module and Spray Angle

The distribution of sprayed droplets and their average velocity were measured by laser plane visualization and particle image velocimetry. This technique allows the capture of two components of the velocity with high spatial resolution in a whole slice of the flow [43]. To visualize the atomization process, instantaneous images were acquired with a Hamamatsu 1,024 × 1,344 pixels 12-bit C4742-95-12 ORCA-ER charge-coupled device (CCD) camera (Hamamatsu Photonics, Shizuoka, Japan), equipped with a Nikon 50 mm F#1.2 lens. The images size were 154 mm × 117.35 mm, yielding a resolution of 114.6 μm/pixel. To freeze the motion, a PILS NdYAG laser from Quanta System (Quanta System, S.p.A., Milan, Italy) was used to illuminate a vertical plane across the center of the spray and generate 6 pulses with a selectable time interval of 30 µs. Images were processed with the CCDPIV computer code developed at the Laboratory for Turbulence Research in Aerospace and Combustion in Monash University (Melbourne, Australia) [44]. Analysis was performed in 32 × 32 pixel windows with 50% overlap, resulting in maps with 82 × 62 velocity vectors.

The mean velocity field of the spray droplets was obtained from 100 instantaneous measurements [45] that have been averaged to determine the angle of the spray cone. This angle was obtained by the ocular location of the limits of the spray in the average image. All the photographs have been taken under the same lighting conditions and have undergone the same renormalization of the light levels captured by the camera.

To eliminate the influence of the operator in the generation of the spray, a pneumatic actuator to drive the manual atomizer was assembled. The duration of the liquid exiting from the nozzle (i.e., the duration of the atomization pulse) varies depending on the pressure supplied to the pneumatic device. This pressure has been adjusted to obtain a reasonable actuation, with a pulse duration of 150 m, as measured from the images.

The spray speed and angle measurements were taken 83 m after the start of the atomization; that is, about 8 m after the mid-pulse. Finally, the commercial atomizers used were manufactured with plastic injection molding, so certain variability in the atomization of each device could be expected. To evaluate the device variability, the actuation of 3 atomizers with distilled water was measured as previously reported [43].

### 2.5. Biocompatibility of Formulations against Keratinocytes

Human immortalized keratinocytes (HaCaT), used as model cells, were grown as monolayers in 75 cm^2^ flasks, incubated with 100% humidity and 5% CO_2_ at 37 °C. They were cultured with phenol red-free Dulbecco’s modified Eagle medium (DMEM) with high glucose, supplemented with 10% fetal bovine serum (FBS) and penicillin and streptomycin. The cells were seeded into 96-well plates at a density of 7.5 × 10^3^ cells/well, and after 24 h of incubation, they were treated for 48 h with the extract in dispersion (water and PEG400) or loaded in vesicles. All the dispersions were properly diluted with the cell medium to reach the desired concentrations (0.03, 0.3, and 3 μg/mL). The possible toxic effects of the formulations towards HaCaT cells has been assessed by measuring the viability by means of the MTT (tetrazolium salt, 3-(4,5-dimethylthiazol-2-yl)-2,5-diphenyltetrazolium bromide) colorimetric test. After 48 h, the medium was removed and MTT (100 µL) was added to each well and incubated at 37 °C for 2/3 h. The formazan crystals formed in viable cells were dissolved in DMSO, and the absorbance was measured at 570 nm with a microplate reader (Synergy 4, BioTek Instruments, AHSI S.p.A, Bernareggio, Italy). All the experiments were repeated at least three times and each time in triplicate. The results are expressed as the percentage of live cells compared to untreated cells (100% viability).

### 2.6. Protective Effect of the Extract in Dispersion or Loaded in Vesicles against Oxidative Stress

HaCaT cells (5 × 10^4^ cells/well) were seeded in 96-well plates with 250 μL of culture medium and incubated at 37 °C for 24 h, then stressed for 4 h with hydrogen peroxide (1:50,000 dilution) and simultaneously exposed to the extract in dispersion or incorporated into the vesicles (final concentrations 3, 0.3, 0.03 μg/mL). Unstressed cells were used as the positive control (100% viability) and hydrogen peroxide-stressed cells, treated with extract-free medium, were used as the negative control. After 4 h of incubation, the medium was removed and the viability of the cells was determined with the MTT colorimetric test, adding 100 μL of reagent in each well. After 2/3 h, the formed formazan crystals were solubilized by adding dimethyl sulfoxide, and their concentration was measured spectrophotometrically at 570 nm as above (Section 2.5).

### 2.7. Statistical Analysis

Results were expressed as mean value ± standard deviation. Statistically significant differences were determined employing variance analysis (ANOVA), and the Tukey’s test and Student’s t-test were performed to substantiate differences between groups using XL Statistics for Windows (XLSTAT BASIC+). The minimum level of significance chosen was *p* < 0.05.

## 3. Results

### 3.1. Vesicle Characterization

**Figure 1:**
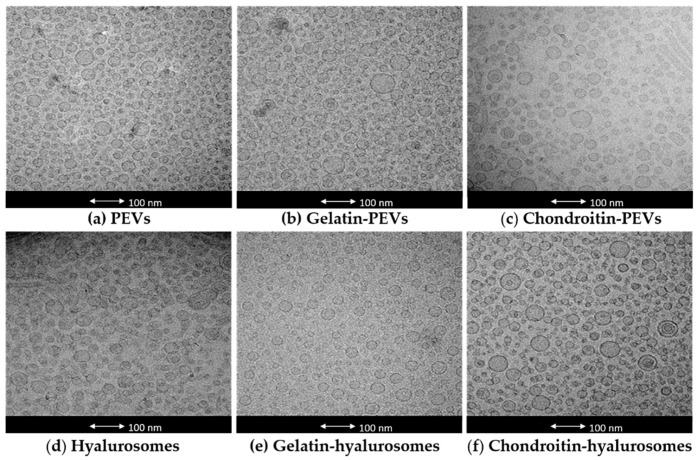
Representative cryo-TEM images of vesicles: (**a**) PEVs; (**b**) gelatin-PEVs; (**c**) chondroitin-PEVs; (**d**) hyalurosomes; (**e**) gelatin-hyalurosomes; (**f**) chondroitin-hyalurosomes.

The actual formation, structure and morphology of *C. halicacabum* extract-loaded vesicles were evaluated by means of cryo-TEM observation. All tested vesicles were mostly small and unilamellar, but some larger vesicles were present as well (Figure 1).

The mean diameter, polydispersity index, zeta potential and entrapment efficiency of the obtained vesicles were measured (Table 1). Empty vesicles were also prepared and characterized to evaluate the effect of the extract on the vesicle assembling. Empty PEVs, chondroitin-PEVs and hyalurosomes were ~106 nm and the others were larger (~150 nm). The loading of the extract did not change the size of PEVs (~100 nm) and gelatin-hyalurosomes (~120 nm, *p* > 0.05 between the mean diameter of empty and corresponding extract-loaded vesicles). On the contrary, the loading of the extract produced an increase of mean diameter of chondroitin-PEVs and chondroitin-hyalurosomes (*p* < 0.05 versus the mean diameter of corresponding empty vesicles) and a reduction of size of the other formulations. The photon correlation spectroscopy only estimates the average size of vesicles by a cumulative intensity-based value, which is weighed according to the scattering intensity of the volume of different particle fractions. In this case, the fraction volume of few larger vesicles present in the dispersions was not clearly represented in the cumulative analysis, causing a small discordance with cryo-TEM images. In addition, the mean diameter of vesicles and its standard deviation were calculated as an average of at least six analyses, which were very repeatable as the standard deviation was very low.

Extract-loaded PEVs were slightly polydispersed (polydispersity index 0.4) and negatively charged (~48 mV). Hyalurosomes were smaller, ~65 nm (*p* < 0.05 versus the mean diameter of PEVs), less polydispersed (polydispersity index 0.3), and negatively charged (~−45 mV) like PEVs (*p* > 0.05 between the value of liposomes and hyalurosomes). The addition of gelatin did not modify the mean diameter and polydispersity index of both PEVs and hyalurosomes, but markedly affected the zeta potential, which became less negative (~−24 mV, *p* < 0.01 versus the value of PEVs and hyalurosomes) due to the positive charge of gelatin on the vesicle surface.

The behavior of chondroitin sulfate was different. Its addition led to an increase of the mean diameter of extract-loaded chondroitin-PEVs (~156 nm, *p* < 0.05 versus the mean diameter of PEVs) and chondroitin-hyalurosomes (~178 nm, *p* < 0.05 versus the mean diameter of hyalurosomes), which were the largest vesicles. On the contrary, chondroitin sulfate allowed the formation of more homogeneous dispersions, as the polydispersity index of chondroitin-PEVs was ~0.1, and that of chondroitin-hyalurosomes ~0.2. Chondroitin did not affect the zeta potential, which might be related to a strong interaction and intercalation of chondroitin within the bilayer, leading to an enlargement of the vesicle diameter.

The entrapment efficiency of the extract in PEVs was very high (~99%) and the addition of the polymers led to a decrease of this parameter (~75%, Table 1). On the contrary, the entrapment efficiency of the extract in hyalurosomes was the lowest (~47%) and the addition of the polymers allowed an increase of the loading, especially using gelatin-hyalurosomes, which reached the same value of PEVs (~90%, *p* > 0.05 versus the values of PEVs).

**Figure 2:**
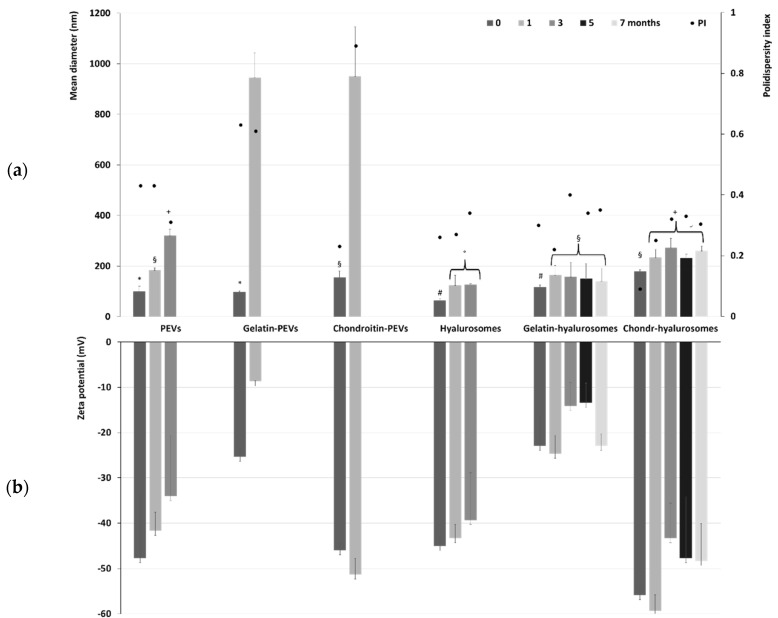
(**a**) Mean diameter, polydispersity index (PI) and (**b**) zeta potential of the extract-loaded PEVs, gelatin-PEVs, chondroitin-PEVs, hyalurosomes, gelatin-hyalurosomes, chondroitin-hyalurosomes, measured over seven months. Data represent the means ± standard deviations of at least six replicates. The same symbol (*, ^§^, ^+^, ^#^, °) indicates the same value.

The long-term stability of the formulations was evaluated by storing the samples for a period of seven months at 4 °C and measuring their physicochemical properties (Figure 2). Temperature of storage is of high importance, as high temperature may promote aggregation and fusion phenomena that are slowed down at lower temperatures such as 4 °C. The mean diameter of PEVs was ~100 nm immediately after preparation, tripled (~320 nm, *p* < 0.05) after three months of storage, and became undeterminable already at the fourth month due to the phase separation and formation of a precipitate of both aggregated vesicles and free extract. The addition of the polymers further reduced the stability of PEVs as the size of gelatin-PEVs and chondroitin-PEVs reached ~950 nm after only one month of storage. After that, it was impossible to measure the size and zeta potential due to the formation of a precipitate mainly composed of aggregated vesicles. Hyalurosomes doubled their diameter after one month of storage (from ~65 nm to ~119 nm, *p* < 0.01 between the two values), and after three months the mean diameter was undetectable due to the formation of large aggregates. The addition of gelatin or chondroitin sulfate to hyalurosomes led to an improvement of the stability of vesicles in dispersion. Indeed, the mean diameter of chondroitin-hyalurosomes increased up to ~260 nm, that of gelatin-hyalurosomes reached ~141 nm after one month of storage, and both remained constant for the following six months. This could indicate that the combination of sodium hyaluronate with fish gelatin or chondroitin sulfate promoted the stability of vesicles in dispersion and facilitated the loading of *C. halicacabum* extract. The zeta potential followed the same trend, remaining coherent and strongly negative during the entire period of the stability check (Figure 2), even if some minor changes could be detected, mainly due to the aggregation and re-assembling of the vesicles in different structures.

### 3.2. Determination of Size Distribution of Sprayed Droplets

The suitability of formulations to be sprayed into the nasal cavity was firstly evaluated by measuring the droplet size distribution. The Food and Drug Administration (FDA) and the European Medicines Agency (EMA) request droplet sizes of greater than 10 µm, to avoid the possibility to being inhaled and reaching the lungs [46]. It is well accepted that nasal local sprays should be composed of particles with a mean size higher than 120 µm to be mainly deposited in the anterior part of the nose [40]. In accordance with the FDA, measurements were performed at a 4 cm and 7 cm distance from the nozzle exit, rotating the pump device 45° in respect to the laser beam [41,47]. The size of 50% of droplets (D50) generated by the used vesicles, except chondroitin-hyalurosomes, were lower than 120 µm (59–89 µm at 4 cm and 47–72 µm at 7 cm), but 90% of droplets were higher than 118 µm (118–196 µm), disclosing the size suitable for the deposition in the anterior region of nose. The size of the droplets generated by chondroitin-hyalurosomes were slightly larger, ~102 µm (D50) and 220 µm (D90) at 4 cm, and 154 µm (D50) and 133 µm (D90) at 7 cm, suggesting a slightly reduced suitability of these formulations to be deposited in the anterior area of nose (Table 2).

### 3.3. Measurements of Spray Plume and Angle 

**Figure 3:**
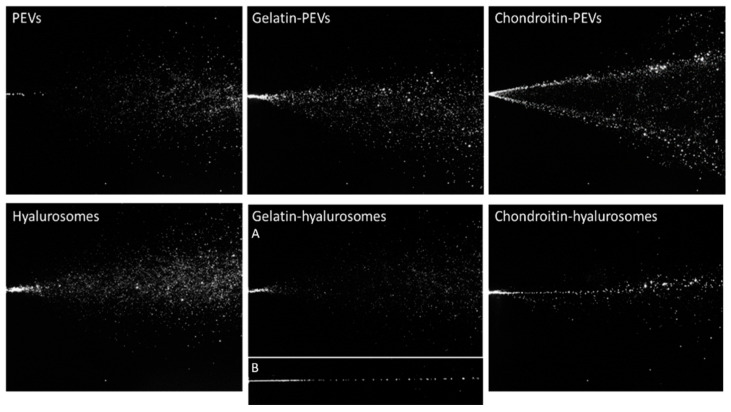
Representative images of instant visualization of plume generated by three consecutive sprays of PEVs; gelatin-PEVs; chondroitin-PEVs; hyalurosomes; (A, B) gelatin-hyalurosomes; and chondroitin-hyalurosomes.

The characteristics of the spray plumes generated by the vesicle dispersions were also measured. To assess the possible variability introduced by pump and nozzle manufacture, the behavior of distilled water sprayed with three different devices has been evaluated and used as a reference, as reported in a previous work [43]. The images of sprayed water established the atomizing ability of the device to compare the results obtained when spraying the formulations of *C. halicacabum* extract loaded in vesicles (Figure 3). 

Dispersions of PEVs and gelatin-PEVs produced a full cone plume, like that of water but less intense and large, while chondroitin-PEVs produced a hollow cone plume, not desirable for our purposes, where the liquid droplets were mainly located in the outer layers and sparsely populated the interior region [43] (Figure 3).

Hyalurosome and gelatin-hyalurosome dispersions generated a full cone plume. Gelatin-hyalurosomes atomization was poorer, and in some cases, a liquid column was generated (Figure 3, gelatin-hyalurosome image B). Chondroitin-hyalurosomes only generated a very narrow cone (almost a liquid column) of large droplets, according to the results obtained by Spraytec^®^, which underlined the formation of large particles having a mean diameter > 100 µm (D50), and containing a small number of particles >200 µm (D90).

The spray angle was calculated by the obtained images of the plume (Table 3). The spray angle of the distilled water cases was ~17.6° as reported in a previous work [37]. The spray cone angle of chondroitin-PEVs and hyalurosomes was the lowest (~19–20°) and similar to that obtained by spraying water. Gelatin-PEVs and gelatin-hyalurosomes generated a large opening plume (~25°), that further increased using PEVs. The plume of the chondroitin-hyalurosomes was not quantified due to the formation of a liquid column. 

#### Measurements of Average Velocity Module 

**Figure 4:**
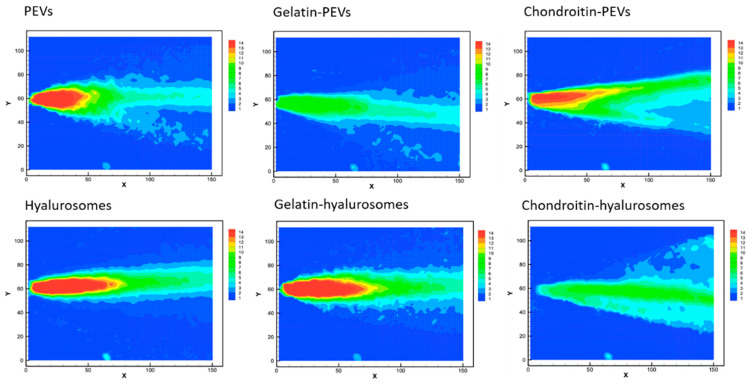
Representative images of average drop speed generated by the spray of the extract loaded PEVs; gelatin-PEVs; chondroitin-PEVs; hyalurosomes; gelatin-hyalurosomes; and chondroitin-hyalurosomes. Red indicates high velocity, yellow indicates intermediate velocity, and green and light-blue indicate low velocity.

The velocity measurements of sprayed vesicles generated, at the atomizer exit, a conical sheet of liquid with decreasing intensity (Figure 4), similar to that of water [40].

The cone generated by PEVs, chondroitin-PEVs, hyalurosomes and gelatin-hyalurosomes had a central part intensely colored in red, indicating high velocity (Figure 4). Indeed, the maximum speed values for PEVs and chondroitin-PEVs were ~13.4 m/s, and those of hyalurosomes and gelatin-hyalurosomes were ~15.1 m/s, thus quite similar in structure and values to those obtained using the water [44]. The maximum velocity of gelatin-PEVs and chondroitin-hyalurosomes was the lowest (~7.7 m/s), as they did not generate a red cone core.

### 3.4. Biocompatibility of Vesicles and Protective Effect against Oxidative Stress Damage

**Figure 5:**
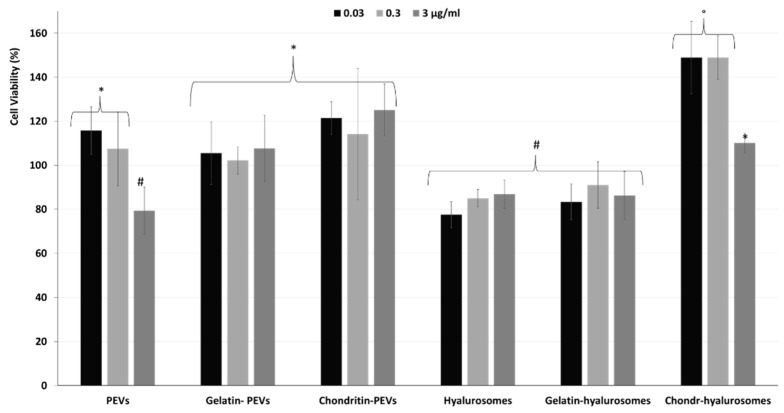

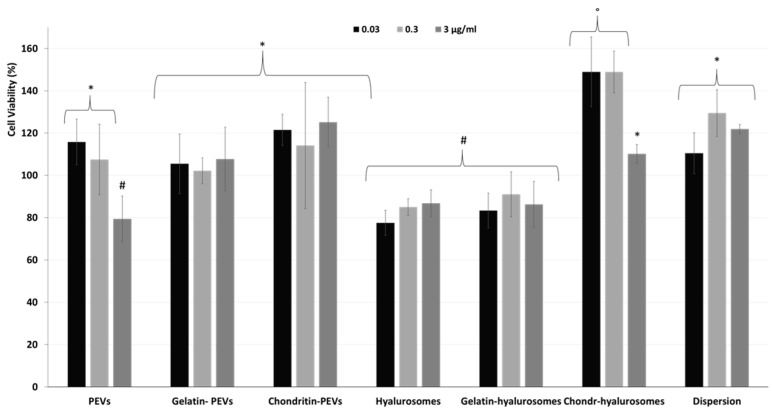
Viability of keratinocytes treated with *C. halicacabum* extract in dispersion or loaded in PEVs; gelatin-PEVs; chondroitin-PEVs; hyalurosomes; gelatin-hyalurosomes; and chondroitin-hyalurosomes, at different dilutions (extract 0.03, 0.3, 3 µg/mL). Data represent the means ± standard deviations of at least three experimental determinations. Each symbol (*, ^#^, °) indicates the same value.

Considering the promising technological properties of the obtained vesicle dispersions, the in vitro biocompatibility and antioxidant activity against keratinocytes were evaluated as well, and compared with that of the extract in dispersion [2,4,5,7,8,9,10,12,48,49]. Firstly, the biocompatibility was monitored incubating the cells with the extract in dispersion or incorporated in the vesicles for 48 h and measuring their viability (Figure 5). The dispersion of the extract was highly biocompatible and even capable of stimulating cell proliferation (viability ~118%, *p* < 0.05 versus the viability of cells treated with chondroitin-hyalurosomes at lower concentrations). The loading of the extract in vesicles differently affected the cell viability as a function of the used carriers and the dilution tested. More specifically, when PEVs were used at the lower concentrations (0.03 and 0.3 µg /mL), the viability was ~115% (*p* > 0.05 versus the viability of cells treated with the dispersion), as found using the extract dispersion. At the highest concentration (3 µg /mL), the cell viability strongly decreased (~80%, *p* < 0.05 versus the values provided by lower concentrations), denoting a low toxicity at this concentration. Using hyalurosomes and gelatin-hyalurosomes, the cell viability was ~80% (*p* < 0.05 versus the viability found using the dispersion), irrespective of the used concentrations. Using gelatin-PEVs and chondroitin-PEVs, the cell viability was ~112% (*p* > 0.05 versus the viability of cells treated with the dispersion or the PEVs at lower concentrations), irrespective of the carrier and concentration used. The addition of chondroitin sulfate in hyalurosomes seems to stimulate cell proliferation, as the viability approached ~150% (*p* < 0.05 versus the other values) when the lower concentrations (0.03 and 0.3 µg/mL) were used. The cell proliferation can be related to the dual effect of sodium hyaluronate and chondroitin, associated with the extract, which can favor the proliferation of keratinocytes, thus suggesting a positive contribution of this formulation in accelerating epithelial regeneration processes [50]. Using these vesicles at the highest concentration (3 µg/mL), the cell viability was lower (~110%) (*p* < 0.05).

**Figure 6:**
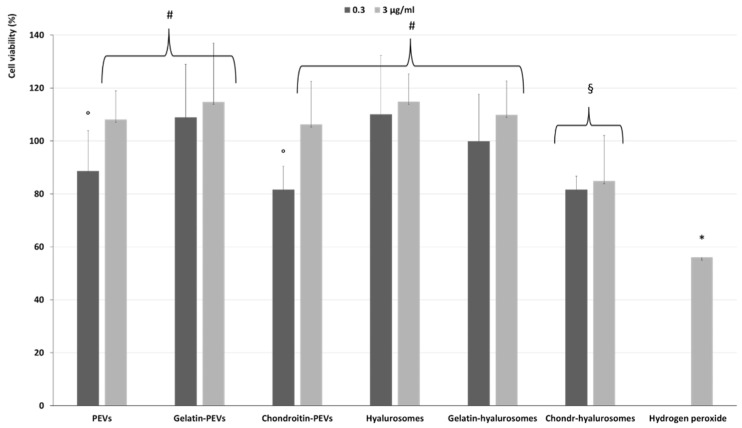

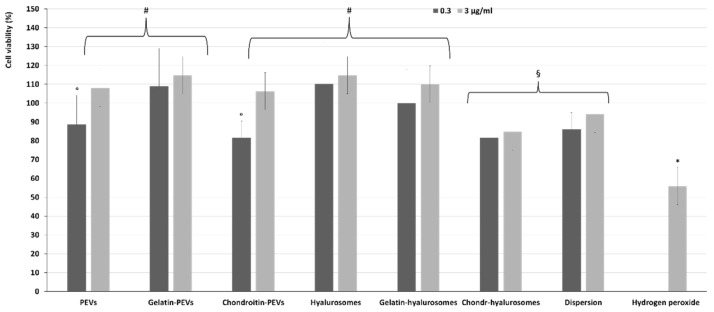
Viability of keratinocytes stressed with hydrogen peroxide and treated with *C. halicacabum* extract-loaded PEVs; gelatin-PEVs; chondroitin-PEVs; hyalurosomes; gelatin-hyalurosomes; chondroitin-hyalurosomes, at different dilutions (extract 0.3, 3 µg/mL). Data represent the means ± standard deviations of cell viability expressed as the percentage of the negative control (100%). Each symbol (*, ^#^, °, ^§^) indicates the same value.

The protective effect of extract-loaded vesicles (0.3 and 3 µg/mL) against oxidative stress damage was evaluated using keratinocytes (Figure 6). The exposure of cells to hydrogen peroxide (untreated cells) reduced the viability up to ~55%, while the addition of the extract in dispersion or loaded in vesicles successfully prevented the cell mortality caused by hydrogen peroxide, thus restoring the healthy conditions. Indeed, the cell viability increased up to ~110% (*p* < 0.01 versus the viability of untreated cells), except when chondroitin-hyalurosomes were used, as these formulations only partially protected the cells, allowing a cell viability of ~85% (*p* < 0.05 versus other values).

## 4. Discussion

Aiming at developing an effective nasal spray based on natural components and capable of reaching the anterior part of nasal mucosa to attenuate nasopharyngeal congestion and rhinitis, *C. halicacabum* extract was loaded into phospholipid vesicles specifically tailored for nasal delivery. *C. halicacabum* extract was selected based on its rich phytocomplex, which contains apigenin, apigenin-7-O-glucuronide, chrysoeriol-7-O-glucuronide, luteolin, luteolin-7-O-glucuronide, saponin, quebrachitol, proanthocyanin, beta-sitosterol and stigmasterol, and its beneficial properties, well known for centuries [1,2,51]. It was loaded in phospholipid vesicles due to their effectiveness in the treatment of rhinitis. Indeed, the simple empty phospholipid vesicles (without bioactives) are currently used to counteract nasal congestion and inflammation, which generally occur in rhinitis, and formulations based on liposomes and hyalurosomes are already available on the market in Europe [16,17]. Definitely, the association of phospholipid vesicles with the extract of *C. halicacabum*, which has anti-inflammatory and antioxidant activities, is expected to provide a synergic effect [19]. To load the highest amount of extract, in a pre-formulation study, several formulations were prepared, testing different amounts and types of phospholipids, water co-solvents, polymers and surfactants. Finally, small and stable vesicles were obtained using 3 mg/mL of the extract of *C. halicacabum* and 92 mg/mL of a commercial mixture of soy phosphatidylcholine (Lipoid S75). The former were hydrated with a mixture of water and PEG400 to prepare PEVs, and with a dispersion of sodium hyaluronate in water and PEG400 to obtain hyalurosomes. PEVs and hyalurosomes were further modified using a commercial cold-water fish gelatin or chondroitin sulfate extracted from catshark cartilages (*Scyliorhinus canicula*) to stabilize the vesicles. Gelatin is widely used in cosmetic, biomedical and pharmaceutical applications [52]. Chondroitin sulfate, similarly to hyaluronic acid, is an eco-sustainable biopolymer that can be obtained from animal wastes and by-products [53].

The obtained vesicles were small sized and negatively charged; the addition of gelatin markedly affected the zeta potential, which became less negative due to the positive charge of gelatin, which is a polyampholyte molecule (~13% positively-charged amino acids, ~12% negatively-charged amino acids and ~11% is hydrophobic amino acids) with cationic and anionic nature as a function of pH and temperature, that may modify its behavior in solution [51,54,55]. Fish gelatin is a cationic type A gelatin, whose isoelectric point is between 7.0 and 9.0 [52,55]. Elzoghby et al. disclosed that the positive charge of gelatin-based nanoparticles is related to the prevalence of NH_3_^+^ groups [50]. In the vesicle surface, gelatin interacted with the negatively-charged phospholipids, leading to an increase in the surface zeta potential, which became less negative.

The addition of chondroitin sulfate mostly affected the mean diameter, forming larger vesicles while the zeta potential remained strongly negative due to its carboxyl groups and sulfo groups, which are probably mainly located on the vesicle surface [56]. The presence of gelatin or chondroitin was not capable to stabilize the PEVs in dispersion, but improved the stability of hyalurosomes, which kept a small size and negative zeta potential for up to seven months.

Considering the small size of the obtained formulations, which are suitable for skin and mucosal delivery, their spray-ability into the nasal cavity was firstly evaluated in vitro by measuring the droplet size distribution, spray angle and droplet velocity [50,57]. Indeed, nasal spray devices commonly generate a cone of aerosolized droplets, called a plume, having a mean size in the range of 10–200 μm, which are recognized as a fine mist able to well deposit in the nasal cavity [37,57,58,59,60,61]. It has been reported that spray pumps that produce droplets >10 µm can ensure its deposition in the nose, and if the droplets are >120 µm, they mostly deposit in the anterior part of the nose, exerting a local effect [40,62]. According to the FDA recommendations, nasal sprays must generate droplets <10 µm to avoid their lung inhalation. In the present study, the size distribution of sprayed droplets was within these parameters, and 90% of droplets (D90) were >118 µm, confirming the suitability of these formulations to be sprayed in the nose, reach the anterior nasal cavity, and exert a local effect. Among all, chondroitin-hyalurosomes were slightly less suitable to deposit in the anterior zone.

The spray plume analysis confirmed the ability of PEVs, gelatin-PEVs, hyalurosomes and gelatin-hyalurosomes to be atomized in fine droplets homogenously distributed in a full cone plume, with an angle ranging from 25 to 30°. Generally, significant changes in the area of deposition have also been reported as a function of plume angle [63]. As the plume angle decreases (narrower plume), an increased deposition in the turbinate region has been reported, but it was also dependent on the droplet size and velocity [62,64]. Indeed, high-speed droplets are mostly deposited in the anterior part of the nose [62]. In this study, PEVs, chondroitin-PEVs, hyalurosomes and gelatin-hyalurosomes generated droplets with maximum speed ~14 m/s, which was equal to that of water, and double if compared to that of gelatin-PEVs and chondroitin-hyalurosomes.

Thus, overall results on sprayed plume underlined that PEVs, hyalurosomes and gelatin-hyalurosomes have the most suitable characteristics for nasal local delivery thanks to their ability to form a full cone of homogeneous droplets having a size of always <196 µm, a cone angle ~27° and high-speed ~14 m/s. The formed droplets can be well deposited in the nasal cavity, which serves as a target site for the treatment of local diseases such as nasal allergic conditions and nasal congestions [65].

In this study, the analysis of technological properties of vesicle dispersions has been associated to the evaluation of their biological efficacy. The biocompatibility of *C. halicacabum* extract in dispersion or loaded in vesicles confirmed that all PEVs and chondroitin-hyalurosomes, along with the extract in dispersion, at lower concentrations (0.03 and 0.3 µg /mL), were not toxic and favored cell proliferation, as the viability of epithelial cells after 48 h of incubation was ≥100%. Differently, using hyalurosomes and gelatin-hyalurosomes the cell viability was ~80%, indicating a weak toxicity of these formulations as previously observed using phospholipid vesicles [66]. Considering that the anti-inflammatory effect of *Cardiospermum halicacabum* extract and phospholipid vesicles were previously demonstrated, to perform a preliminary biological evaluation of formulations, their ability to protect the tissues from the oxidative damages was also evaluated. Indeed, oxidative stress can activate a variety of transcription factors, which lead to the differential expression of some genes involved in inflammatory pathways. The inflammation triggered by oxidative stress is the cause of many chronic diseases [67]. All the formulations, irrespective to their composition, inhibited the damaging effect of hydrogen peroxide in the cells at the intermediate non-toxic concentration used (0.3 µg/mL), counteracting their damages and death [68]. Their effectiveness can be mainly connected to the ability of vesicles to interact with cells favoring the internalization of the bioactives contained in the extract, which are mainly flavonoids and polyphenols capable of chelating iron and scavenging free radicals [69,70]. The beneficial effect of extract loaded in chondroitin-hyalurosomes was lower than that of extract in dispersion or loaded in other vesicles, such as PEVs, hyalurosomes and gelatin-hyalurosomes, which also disclosed the better spray-ability suggesting an effective deposition in the nasal cavity and improved biological performances. Among these formulations, considering the overall results comprehensive of physicochemical, spray-ability and biological parameters, gelatin-hyalurosomes seemed to be the most promising and effective dispersion for the treatment of nasal disorders connected with oxidative stress.

## 5. Conclusions

The evaluation of physicochemical, technological and biological properties of *C. halicacabum* extract-loaded vesicles indicated that the prepared formulations, especially PEVs, hyalurosomes and gelatin-hyalurosomes, are suitable to be atomized and deposited in the anterior nasal cavity, where they can exert local activity against congestion and rhinitis. In addition, they are able to protect the epithelial cells from oxidative stress, avoiding local tissue damages. Gelatin-hyalurosomes showed long-term stability with storage and seemed to be the most promising formulation for nasal delivery, thus encouraging their possible application for the treatment of nasopharyngeal congestion and rhinitis associated with oxidative stress. Alternatively, the other formulations, like PEVs and hyalurosomes, which could not keep their physicochemical properties during storage, could be further stabilized by using other additives.

## Figures and Tables

**Table 1:** Mean diameter (MD), polydispersity index (PI), zeta potential (ZP) and entrapment efficiency (EE) of empty and extract-loaded PEVs, gelatin-PEVs, chondroitin-PEVs, hyalurosomes, gelatin-hyalurosomes, chondroitin-hyalurosomes. Data represent the mean ± standard deviation of at least six replicates. The same symbol (*, ^§^, ^+^, ^#^, °) indicates the same value (*p* < 0.05).

	MD (nm)	PI	ZP (mV)	EE (%)
Empty PEVs	* 92 ± 2	0.23	−67 ± 4	
Empty gelatin-PEVs	^+^ 175 ± 25	0.31	−55 ± 7	
Empty chondroitin-PEVs	^#,^* 117 ± 30	0.43	−13 ± 3	
Empty hyalurosomes	* 92 ± 3	0.45	−21 ± 10	
Empty gelatin-hyalurosomes	^#^ 129 ± 3	0.13	−30 ± 12	
Empty chondroitin-hyalurosomes	^§^ 154 ± 13	0.20	−44 ± 10	
PEVs	* 100 ± 20	0.43	−48 ± 14	* 99 ± 16
Gelatin-PEVs	* 98 ± 4	0.63	−25 ± 2	° 73 ± 36
Chondroitin-PEVs	^§^ 156 ± 23	0.23	−46 ± 1	° 75 ± 10
Hyalurosomes	° 65 ± 6	0.26	−45 ± 2	47 ± 13
Gelatin-hyalurosomes	^#^ 118 ± 8	0.30	−23 ± 6	* 93 ± 57
Chondroitin-hyalurosomes	^+^ 178 ± 6	0.09	−55 ± 2	° 65 ± 9

**Table 2:** Average diameter of droplets generated by spraying *C. halicacabum* extract-loaded PEVs, gelatin-PEVs, chondroitin-PEVs, hyalurosomes, gelatin-hyalurosomes, and chondroitin-hyalurosomes, and measured using Spraytec^®^. Mean values ± standard deviations of three measurements were reported. The same symbol (*, ^§^, ^+^, ^#^, °,^@^) indicates the same value.

	4 cm				7 cm			
	D10 (µm)	D50 (µm)	D90 (µm)	SPAN (µm)	D10 (µm)	D50 (µm)	D90 (µm)	SPAN (µm)
PEVs	29 ± 2	*^,+^ 63 ± 18	^§^ 130 ± 46	2 ± 1	29 ± 2	^+^ 57 ± 1	^@^ 173 ± 53	3 ± 1
Gelatin-PEVs	31 ± 3	^#^ 89 ± 10	^@^ 185 ± 43	3 ± 1	31 ± 1	* 66 ± 6	^@^ 168 ± 50	4 ± 1
Chondroitin-PEVs	33 ± 2	° 69 ± 4	^§^ 131 ± 6	1 ± 0	30 ± 1	* 47 ± 20	^§^ 118 ± 2	1 ± 0
Hyalurosomes	27 ± 1	*^,+^ 59 ± 8	^§^ 154 ± 49	3 ± 1	33 ± 1	* 61 ± 2	^@^ 162 ± 76	4 ± 2
Gelatin-hyalurosomes	30 ± 1	^#^ 81 ± 9	^@^ 171 ± 27	3 ± 1	33 ± 2	° 72 ± 2	^@^ 196 ± 58	3 ± 1
Chondroitin-hyalurosomes	30 ± 3	102 ± 48	220 ± 27	3 ± 1	38 ± 5	^§^ 154 ± 129	^§^ 133 ± 30	4 ± 2

**Table 3:** High speed values and spray angle measured from the plume generated by *C. halicacabum* extract-loaded PEVs; gelatin-PEVs; chondroitin-PEVs; hyalurosomes; gelatin-hyalurosomes; and chondroitin-hyalurosomes.

	High-Speed (M/S)	Spray Angle (°)
PEVs	13.9	29.7
Gelatin-PEVs	8.0	24.5
Chondroitin-PEVs	13.1	19.2
Hyalurosomes	15.2	20.7
Gelatin-hyalurosomes	15.1	25.4
Chondroitin-hyalurosomes	7.5	-

## Data Availability

Data supporting reported results is not available.

## Sample Availability

Samples of the compounds are not available from the authors.
